# Asynchronous Evolution of Nanoporous Silver on Dual-Phase Ag–Sn Alloys by Potentiostatic Dealloying in Hydrochloric Acid Solution

**DOI:** 10.3390/nano9050743

**Published:** 2019-05-14

**Authors:** Yulin Yang, Zhenhua Dan, Yongfeng Liang, Ying Wang, Fengxiang Qin, Hui Chang

**Affiliations:** 1 College of Materials Science and Engineering and Tech Institute for Advanced Materials, Nanjing Tech University, Nanjing 210009, China; dan9506@gamil.com (Y.Y.); ch20006@njtech.edu.cn (H.C.); 2 State Key Lab of Advanced Metals and Materials, University of Science and Technology Beijing, Beijing 110083, China; yfliang@skl.ustb.edu.cn; 3 State Key Laboratory of Metal Material for Marine Equipment and Application, Anshan 114021, China; yingwang2335@163.com; 4 School of Materials Science and Engineering, Nanjing University of Science and Technology, Nanjing 210094, China

**Keywords:** nanoporous silver, dual-phase Ag–Sn alloys, potentiostatic dealloying, surface diffusivity, potentiodynamic polarization behavior

## Abstract

Evolution behavior of the nanoporous architectures has been investigated via potentiostatic electrochemical dealloying of dual-phase Ag*_x_*Sn_100−*x*_ (*x* = 20, 30, 40 at.%) alloys, which consist of β-Sn and ε-Ag_3_Sn phases with different volume fractions in 1.2 M HCl solution. The results show that the open-circuit potentials and corrosion potentials of dual-phase Ag–Sn alloys are determined by the less noble β-Sn phases rather than chemical compositions of the Ag–Sn precursor alloys. The potentiodynamic polarization curves show that the anodic dissolution of Ag–Sn alloys is divided into two stages including the first preferential dissolution of β-Sn phases and secondary dealloying of ε-Ag_3_Sn phases, which is associated with the order of the nanoporous evolution. Nanoporous silver (NPS) can be fabricated by potentiostatic dealloying of dual-phase Ag–Sn alloys in HCl solution. The dealloying of two phases is asynchronous: The less noble β-Sn phases are preferentially etched to generate the larger pores, and then the more noble ε-Ag_3_Sn phases are dealloyed to form the finer nanoporous structure. The significant surface diffusion of Ag adatoms at the applied potential higher than the pitting potential of ε-Ag_3_Sn phases during the dealloying results in the coarsening of nanoporous ligaments with a time dependence of *d*(*t*) ∝ *t*^0.1^. The fractions and the difference in electrochemical stabilities of the β-Sn and ε-Ag_3_Sn phases in dual-phase Ag*_x_*Sn_100−*x*_ (*x* = 20, 30, 40 at.%) precursor alloys determines the final nanoporous structure.

## 1. Introduction

Nanoporous metals (NPMs) with high specific surface areas have attracted great attention in a wide variety of applications including catalysis, sensors, actuators, and surface-enhanced Raman scattering (SERS) [1,2,3,4,5,6,7,8]. Chemical or electrochemical dealloying referring to the selective dissolution of binary or multi-component alloys has commonly been used to fabricate NPMs [9,10,11,12,13,14,15]. The dealloying method can simply and flexibly modulate the microstructure and length scale of NPMs via designing the chemical compositions and microstructures of the precursor alloys [9,10], changing the dealloying solution [11,12], dealloying time [13] and temperature [14], and using post-dealloying treatments [15]. As the dealloying can be affected by many factors, the mechanism of dealloying has been discussed. Forty and Durkin [16,17] constructed a terrace-ledge-kink model to explain the selective dissolution of Ag element and surface diffusion of Au element during the dealloying of Ag–Au alloys. Erlebacher et al. [18] have succeeded in unveiling nanoporosity evolution during dealloying of Ag–Au alloys by a kinetic Monte Carlo model. It is generally accepted that the formation of NPMs during dealloying involves selective dissolution of the less noble metal atoms or phases and simultaneous arrangements of the more noble metal atoms to form the ligaments under the driving force of surface diffusion [9,11,18]. The underlying dealloying mechanism of precursor alloys with multi-phase microstructures is so far ambiguous and urgently needs further investigation. Numerous studies have revealed that the initial microstructure of precursor alloys has an important influence on the final nanoporous structures of NPMs obtained by dealloying [10,19,20]. For example, nanoporous nickel (NP-Ni) fabricated from the Mn_62_Ni_38_ precursor alloys has a bimodal structure because of the different dealloying behavior of the coexisting NiMn and NiMn_2_ intermetallics during the electrochemical dealloying [21]. Therefore, exploring the dealloying behavior of related precursor alloy systems would be meaningful to further understand the dealloying mechanism, and can be used to design the microstructure of the precursor alloys and predict the final nanoporous structures. Early studies on dealloying behavior and mechanism mainly focused on the binary precursor alloys with a single-phase solid state, such as Ag–Au [14,15,16,17,18,22,23,24], Mn–Cu [25], and Cu–Pt [26]. Those precursors are generally considered to be advantageous for obtaining ideal and isotropic bi-continuous nanoporous structures. In addition, amorphous precursor alloys, such as Ti–Cu [12], Mg–Cu–Y [11,13], and Pd–Cu–Ni–P [20], have been introduced into dealloying because they are homogenous in chemical compositions and microstructure without defects such as grain boundary. The fine and uniform nanoporous copper (NPC) and nanoporous palladium (NP-Pd) have been fabricated from amorphous precursors mentioned above. However, it should be noted that many alloy systems consist of two or more phases in nanocrystalline or coarsening crystalline alloys in the ordinary solidification conditions. Several dual-phase or multi-phase alloys, such as Al–Au [19,27], Al–Cu [10], and Zn–Cu [28], have been successfully used to produce corresponding NPMs. The nanoporous structures obtained from the aforementioned precursors are usually affected by the disparities in chemical compositions and structure between coexistent phases, especially intermetallics, and this leads to the complexity in dealloying process. For example, Liu [29] has found the structural evolution from nanoporous Mg_2_Cu intermetallics (NP-Mg_2_Cu) to the NPC with bimodal or unimodal nanopore distributions during the dealloying of dual-phase Mg–Cu alloys. Thus, it is necessary to determine the dealloying behavior of each coexistent phase in these dual-phase or multi-phase precursor alloys.

Nanoporous silver (NPS) is an important kind of NPMs due to its potential applications in molecular adsorptions, ion exchange, medical materials, and heterogeneous catalysis [30]. Recently, several alloy systems including crystalline Al–Ag [27], Mg–Ag [9], Zn–Ag [30], and amorphous Ca–Ag [31], Ag–Cu–Si [32] have been successfully developed to fabricate NPS through chemical or electrochemical dealloying. Compared to chemical dealloying, electrochemical dealloying has certain advantages in shorting dealloying duration, modulating nanoporous structures by potential or current control, and etching some inert intermetallics that are not easily dealloyed by chemical dealloying methods [10,15,20,21,26,28,30].

In this work, the dual-phase Ag–Sn alloys have been chosen to fabricate the NPS through potentiostatic electrochemical dealloying in HCl solution. The electrochemical properties of the Ag–Sn alloys have been discussed based upon open-circuit potential measurements and potentiodynamic polarization analysis. The dealloying behavior of dual-phase Ag–Sn alloys has been investigated via observing the microstructure evolution, constitution of the phases and chemical compositions, and change in ligament/pore size during electrochemical dealloying. In addition, the coarsening mechanism of nanoporous ligaments has been discussed from the perspective of surface diffusion of Ag adatoms, and the surface diffusivity of Ag adatoms during dealloying has also been evaluated.

## 2. Experimental Procedure

The Ag*_x_*Sn_100−*x*_ (*x* = 20, 30, 40 at.%) alloys with nominal compositions of Ag_20_Sn_80_, Ag_30_Sn_70_, and Ag_40_Sn_60_ (at.%) were prepared by arc-melting pure Ag (99.99 wt.%) and Sn (99.99 wt.%) under a high-purity argon atmosphere. The ribbons with a thickness of 20 μm and a width of 6 mm were produced from Ag–Sn alloys by the single roller melt spinning technique in an argon atmosphere. The reference ε-Ag_3_Sn intermetallic ribbons were also produced by the same process mentioned above. Single-phase β-Sn (99.99 wt.%) and fcc Ag (99.99 wt.%) foils were purchased from Beijing Central New Metallic Mateials Technology Co. Lit. (Beijing, China).

Electrochemical experiments were performed in a conventional three electrode cell in 1.2 M HCl solution at room temperature. The Ag–Sn ribbons were used as the work electrode, and an Ag/AgCl electrode in 3.33 M KCl solution as the reference electrode and the Pt plate as the counter electrode. All the potential was referred to the Ag/AgCl (3.33 M KCl) electrode unless otherwise stated. The electrochemical properties of Ag–Sn alloys and reference foils were characterized by the measurements of open circuit potentials and potentiodynamic polarization curves. The scan rate for potentiodynamic polarization was 1 mV s^−1^. Potentiostatic dealloying was performed at different applied potentials for different times to study the dealloying mechanism and fabricate NPS. The phase constitution and microstructure of as-spun and as-dealloyed ribbons was confirmed by an X-ray diffractometer (XRD, Rigaku, RINT-4200, Tokyo, Japan) and a transmission electron microscope (TEM, JEOL, JEM-2100F, Tokyo, Japan). The mean sizes of nanopores and ligaments were obtained by measuring over 125 sites on the SEM images by using single-chord method. The TEM samples were prepared by ion milling method. The surface morphology and composition of the as-dealloyed ribbons was observed by a scanning electron microscope (SEM, FEI, QUANTA 250 FEG, Hillsboro, OR, USA) with an energy dispersive X-ray analyzer (EDX, FEI, QUANTA 250 FEG, Hillsboro, OR, USA).

## 3. Results

### 3.1. Effect of Phase Constitutions of Ag–Sn Precursor Alloys on the Electrochemical Behavior 

**Figure 1:**
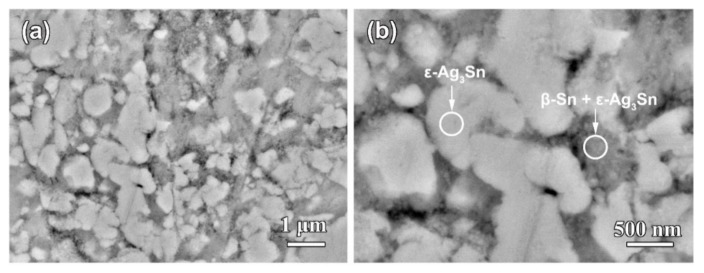
Low-magnified (**a**) and high-magnified (**b**) top-view backscattered electrons (BSE) images of Ag_40_Sn_60_ alloy ribbons.

**Figure 2:**
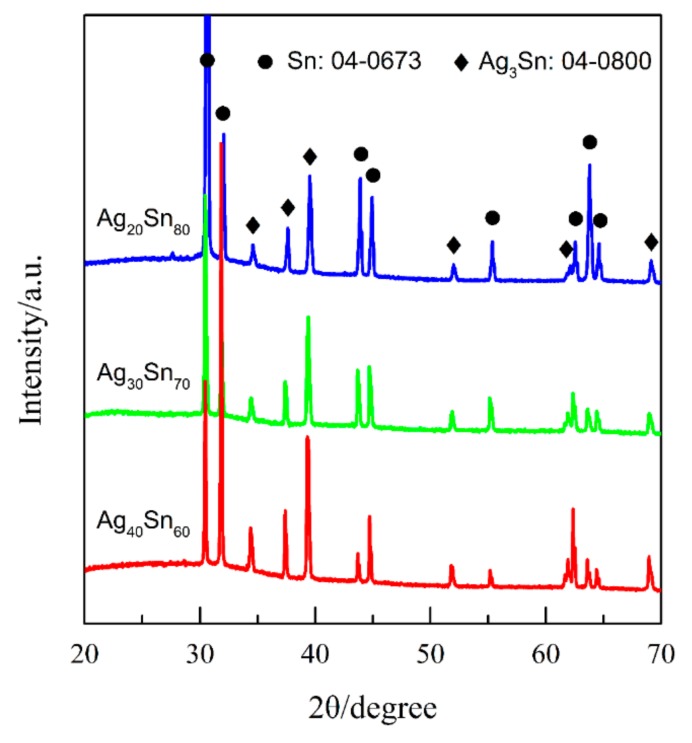
XRD patterns of as-spun Ag_20_Sn_80_, Ag_30_Sn_70_, and Ag_40_Sn_60_ ribbons.

Figure 1 shows the top-view back-scattering electron images of Ag_40_Sn_60_ ribbons. It is found that two phases coexist in the matrix, where the camber-like Ag-rich phase is enveloped by the Sn-rich phase. XRD patterns of as-spun Ag_20_Sn_80_, Ag_30_Sn_70_, and Ag_40_Sn_60_ ribbons are shown in Figure 2. The result in Figure 1 and Figure 2 indicate that Ag–Sn alloys are composed of two phases including a tetragonal β-Sn (JCPDS 04-0673; Space group: *I*4_1_*/amd*) phase and an orthorhombic ε-Ag_3_Sn (JCPDS 04-0800; Space group: *pmmn*) intermetallic phase. According to the binary Ag–Sn phase diagram [33], the ε-Ag_3_Sn phase and β-Sn/ε-Ag_3_Sn eutectic phase will appear in all of Ag_20_Sn_80_, Ag_30_Sn_70_, and Ag_40_Sn_60_ alloys. Therefore, the Ag-rich and Sn-rich phases in Figure 1 are ε-Ag_3_Sn phase and β-Sn/ε-Ag_3_Sn eutectic phases, respectively. According to the Ag–Sn phase diagram, the β-Sn phases are only able to form when the Sn concentration exceeds 99.91 at.%, which indicated that the volume fraction of the β-Sn phases is very limited [33]. In addition, the molar ratios of ε-Ag_3_Sn phases and β-Sn/ε-Ag_3_Sn eutectic phases are confirmed as about 1.03, 0.58, and 0.29 in Ag_40_Sn_60_, Ag_30_Sn_70_, and Ag_20_Sn_80_ alloys on the basis of Ag–Sn phase diagram [33], respectively. Therefore, the volume fractions of ε-Ag_3_Sn phases in the as-spun Ag*_x_*Sn_100−*x*_ (*x* = 20, 30, 40 at.%) ribbons are considered to increase with the increase of Ag concentrations.

**Figure 3:**
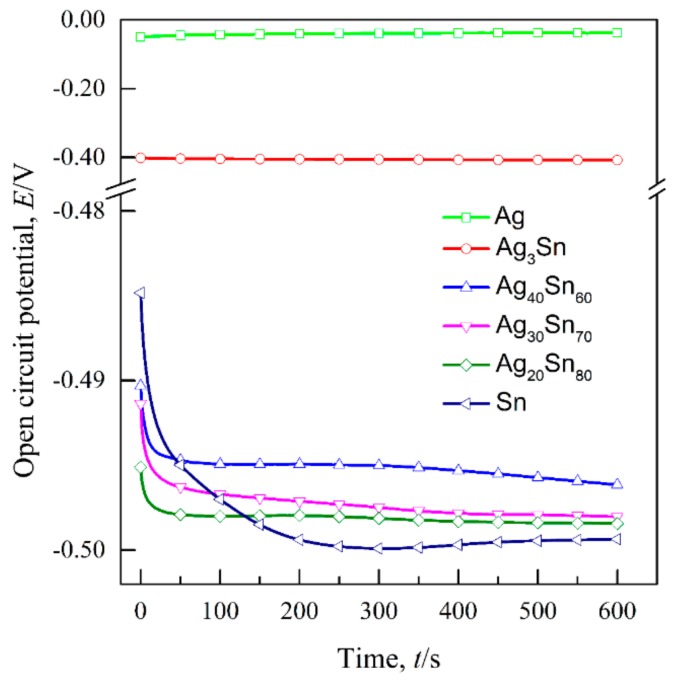
Open-circuit potential of single-phase β-Sn and Ag foils, ε-Ag_3_Sn intermetallic and dual-phase Ag_20_Sn_80_, Ag_30_Sn_70_, Ag_40_Sn_60_ ribbons in 1.2 M HCl solution.

**Figure 4:**
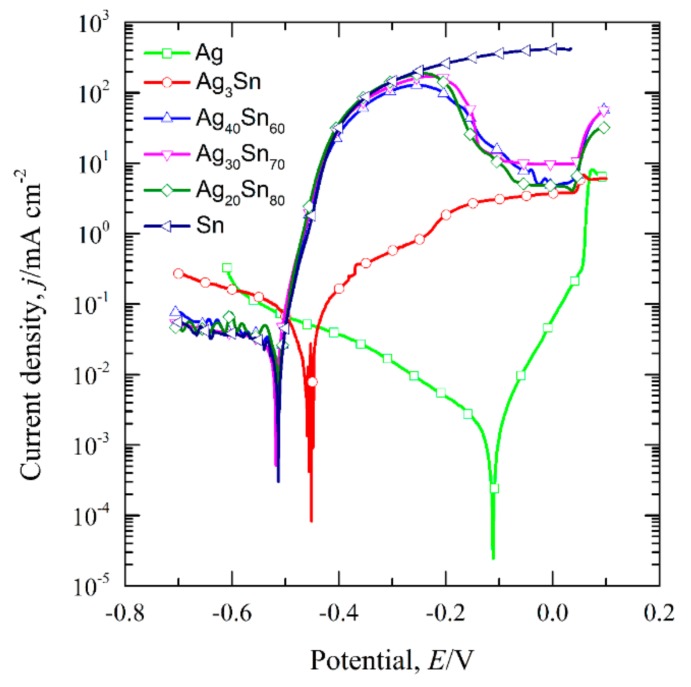
Potentiodynamic polarization curves of dual-phase Ag_20_Sn_80_, Ag_30_Sn_70_, Ag_40_Sn_60_ ribbons and reference materials of single-phase β-Sn and Ag foils, ε-Ag_3_Sn intermetallic ribbons in 1.2 M HCl solution. Scan rate: 1 mV s^−1^.

The transient curves of the open-circuit potential (E_ocp_) of the dual-phase Ag–Sn alloys, single-phase ε-Ag_3_Sn, β-Sn, and Ag in 1.2 M HCl solutions are shown in Figure 3. The E_ocp_ were determined by calculating the average values of potentials during 300–600 s and are given in Table 1. It can be seen that the E_ocp_ of Ag_20_Sn_80_, Ag_30_Sn_70_, and Ag_40_Sn_60_ alloys are close to that of β-Sn, and are about 90 mV and 460 mV lower than that of ε-Ag_3_Sn and Ag, respectively. Figure 4 shows the potentiodynamic polarization curves of as-spun alloys and fiols in 1.2 M HCl solution. The corrosion potential (E_corr_) was measured by the Tafel method [34]. All the E_corr_ are also listed in Table 1. The E_corr_ of ε-Ag_3_Sn and Ag are about 60 mV and 400 mV higher than those of dual-phase Ag–Sn alloys and β-Sn, respectively. As shown in Figure 4, a peak in the potential range of −0.51 V~−0.10 V and a plateau at −0.10 V~0.04 V were observed on the polarization curves of Ag_20_Sn_80_, Ag_30_Sn_70_, and Ag_40_Sn_60_ alloys. The currents increased again at potentials of above 0.04 V. The peak current densities at about −0.24 V were 187 mA cm^−2^, 172 mA cm^−2^, and 129 mA cm^−2^ for Ag_20_Sn_80_, Ag_30_Sn_70_, and Ag_40_Sn_60_ alloys, respectively. The current plateaus were maintained in the range of 5~10 mA cm^−2^. The polarization curves of Sn foils coincided with those of Ag–Sn alloys at potentials of below −0.24 V, but the current increased continuously at potentials above −0.24 V. The currents of ε-Ag_3_Sn phases went through a slower increase in the potential range of −0.46 V~0.04 V, followed by a sharp increase at potentials above 0.04 V. As to the polarization curve of Ag foils, a less obvious passivation region appeared in the potential range of −0.11 V~0.04 V. It was also observed in the previous reports [27]. The present results show that the open-circuit potentials and corrosion potentials are mainly determined by the less noble β-Sn phase in dual-phase Ag–Sn alloys, rather than the alloy compositions.

### 3.2. Morphological Characteristics of Nanoporous Silver after Potentiostatic Dealloying of Dual-Phase Ag–Sn Alloys

**Figure 5:**
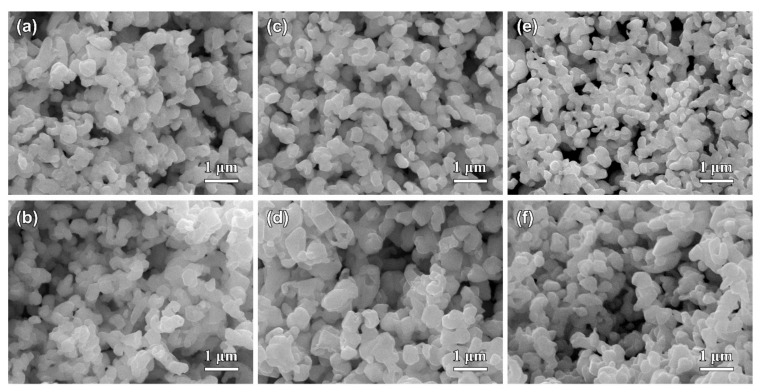
The top-view (**a**,**c**,**e**) and cross-sectional (**b**,**d**,**f**) SEM images of Ag_20_Sn_80_ (**a**,**b**), Ag_30_Sn_70_ (**c**,**d**), and Ag_40_Sn_60_ (**e**,**f**) ribbons after dealloying at −50 mV in 1.2 M HCl solution.

**Figure 6:**
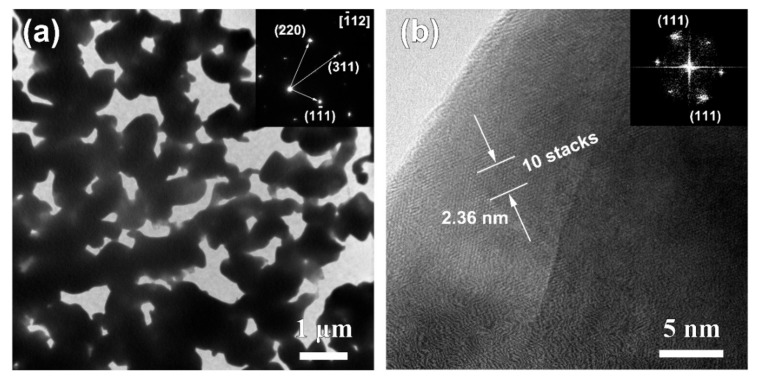
Bright-field TEM image (**a**), high-resolution TEM image (**b**) of Ag_40_Sn_60_ ribbons after dealloying of 5529 s at −50 mV in 1.2 M HCl solution. Insets in (**a**,**b**) are selected area diffraction pattern and FFT pattern.

Figure 5 shows the top-view and cross-sectional SEM images of Ag_20_Sn_80_, Ag_30_Sn_70_, and Ag_40_Sn_60_ ribbons after potentiostatic dealloying at potential of −50 mV in 1.2 M HCl solution. The dealloying conditions are given in Table 2. As shown in Figure 5, bi-continuous nanoporous structure with the open-cell pores and ligaments, typical of nanoporous metals [9,27,30], formed in all as-dealloyed ribbons. The mean sizes of the ligaments and pores are summarized in Table 2. The nanoporous structures of as-dealloyed Ag_20_Sn_80_, Ag_30_Sn_70_, and Ag_40_Sn_60_ ribbons had a ligament size of 182 ± 38 nm, 213 ± 33 nm, and 252 ± 32 nm and a pore size of 301 ± 49 nm, 249 ± 39 nm, and 222 ± 54 nm. In addition, the residual Sn in as-dealloyed ribbons were detected by EDX. The EDX results indicated the residual Sn were 2.60 at.%, 0.81 at.%, and 1.17 at.% in as-dealloyed Ag_20_Sn_80_, Ag_30_Sn_70_, and Ag_40_Sn_60_ ribbons, respectively. XRD patterns of dual-phase Ag_20_Sn_80_, Ag_30_Sn_70_, and Ag_40_Sn_60_ ribbons after dealloying is similar to that shown by the lowest one in Section 3.3. The strong diffraction peaks at 2θ = 38.1°, 44.3°, and 64.4° were assigned to Ag (111), (200), and (220), while a weak diffraction peak at 2θ = 39.5° was attributed to ε-Ag_3_Sn (111). The present EDX and XRD results show that the as-dealloyed ribbons are dominantly composed of fcc Ag phase and trace undealloyed ε-Ag_3_Sn phase. The residual ε-Ag_3_Sn phase is expected to be trapped inside the Ag ligaments and cannot be dealloyed [35,36]. The nanoporous structure obtained by dealloying Ag_40_Sn_60_ ribbon at −50 mV was characterized by TEM as shown in Figure 6. As shown in Figure 6a, the nanoporous structure had a bi-channel structure with ligaments and pores. The SAED pattern (inset of Figure 6a) indicates that the selected area in the ligaments is in a single crystalline state and can be indexed to the fcc Ag viewed along the [$\bar{1}$12] zone axis. The HRTEM image in Figure 6b shows that the lattice fringes extended throughout the whole ligaments, further indicating the existence of the single crystalline phase in the ligaments. The interplanar distance between the adjacent fringes was confirmed to be about 2.36 nm for 10 stacks of the lattice panels of fcc Ag (111). The bright diffraction spots in the fast Fourier transform (FFT) pattern (inset of Figure 6b) are also attributed to fcc Ag (111).

**Figure 7:**
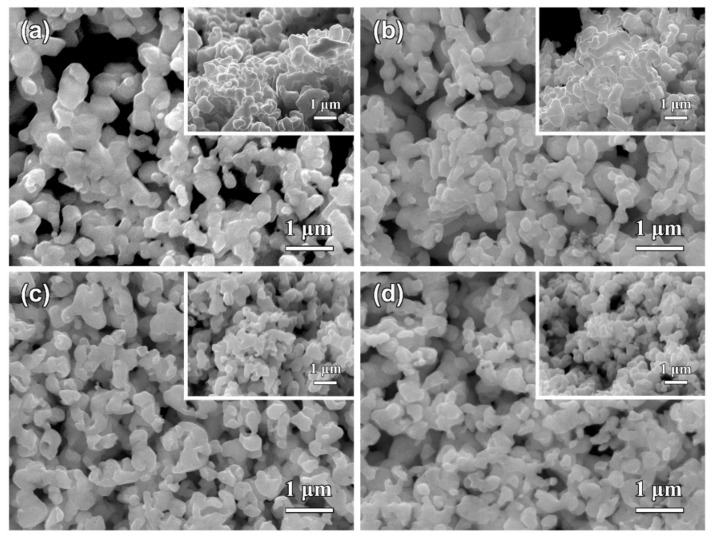
The top-view SEM images of Ag_40_Sn_60_ ribbons after dealloying at 0 mV (**a**), −25 mV (**b**), −75 mV (**c**), and −100 mV (**d**) in 1.2 M HCl solution. Insets in (**a**–**d**) showing the cross-sectional SEM images of as-dealloyed ribbons.

The SEM images of Ag_40_Sn_60_ ribbons after potentiostatic dealloying at potential of 0, −25, −75, and −100 mV in 1.2 M HCl solution are shown in Figure 7. The similar nanoporous structures can be observed in the as-dealloyed ribbons. The ligament sizes of nanoporous structures obtained by dealloying at 0, −25, −75, and −100 mV were 306 ± 50 nm, 278 ± 55 nm, 210 ± 32 nm, and 190 ± 29 nm, and the pore sizes were 295 ± 42 nm, 250 ± 34 nm, 190 ± 20 nm, and 165 ± 39 nm. The increase of the ligament sizes with increasing applied potential is attributed to coarsening by the large driving force from the high applied dealloying potentials and concentrated HCl solutions. The EDX results demonstrate that all Sn was leached away for the ribbon dealloyed at 0 mV and the residual Sn were 0.29 at.%, 1.22 at.%, and 1.33 at.% in ribbons dealloying at −25, −75, and −100 mV, respectively. Moreover, the applied potentials have a significant influence on the dealloying duration, which was much different at various applied potential, as listed in Table 2. For example, the dealloying duration was 36745 s at the potential of −100 mV but was only 1924 s at the potential of 0 mV. Therefore, it is of a great importance to select the suitable applied potential to prepare NPS by potentiostatic dealloying dual-phase Ag–Sn precursor alloys. The higher applied potential can effectively shorten the dealloying time but is not conducive to the formation of smaller ligaments and pores.

### 3.3. Nanoporous Structures and Phase Changes during Potentiostatic Dealloying

**Figure 8:**
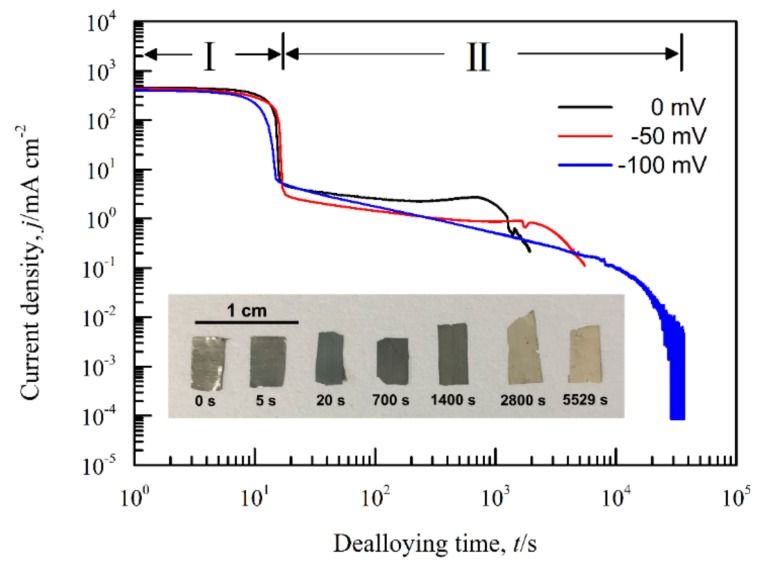
Chronoamperometric curves of Ag_40_Sn_60_ alloy ribbons at 0 mV, −50 mV, and −100 mV in 1.2 M HCl solution. Insets show the optical photos of Ag_40_Sn_60_ ribbons before and after dealloying at −50 mV for different times in 1.2 M HCl solution.

**Figure 9:**
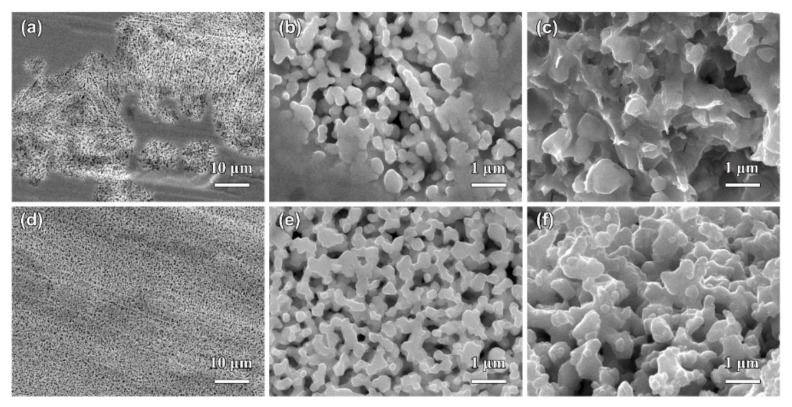
The top-view (**a**,**b**,**d**,**e**) and cross-sectional (**c**,**f**) SEM images of Ag_40_Sn_60_ ribbons after dealloying at −50 mV for 5 s (**a**–**c**) and 20 s (**d**–**f**) in 1.2 M HCl solution.

**Figure 10:**
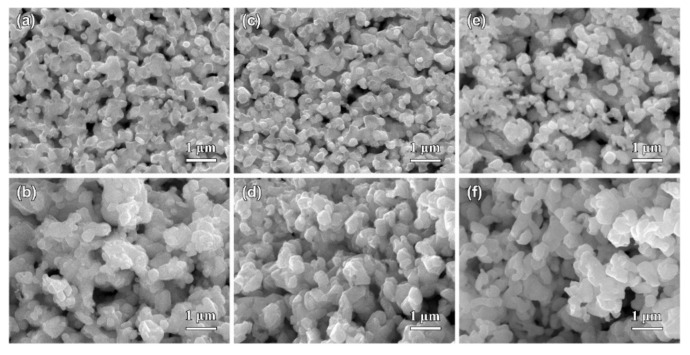
The top-view (**a**,**c**,**e**) and cross-sectional (**b**,**d**,**f**) SEM images of Ag_40_Sn_60_ ribbons after dealloying at −50 mV for 700 s (**a**,**b**), 1400 s (**c**,**d**), and 2800 s (**e**,**f**) in 1.2 M HCl solution.

**Figure 11:**
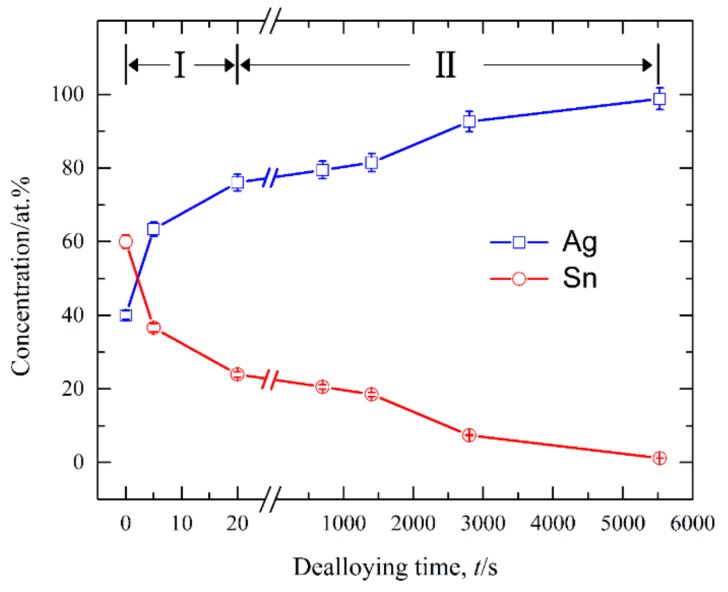
The change of the atomic ratios of Ag and Sn elements after dealloying of Ag_40_Sn_60_ ribbons for different duration times at −50 mV in 1.2 M HCl solution.

**Figure 12:**
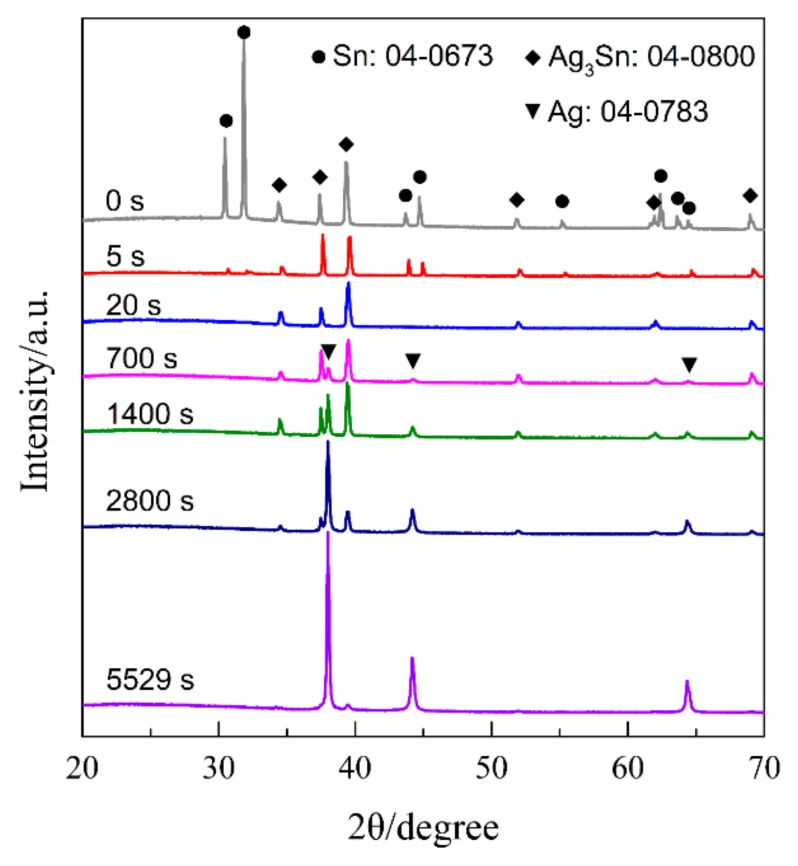
XRD patterns of Ag_40_Sn_60_ ribbons after dealloying at −50 mV for different times in 1.2 M HCl solution.

**Figure 13:**
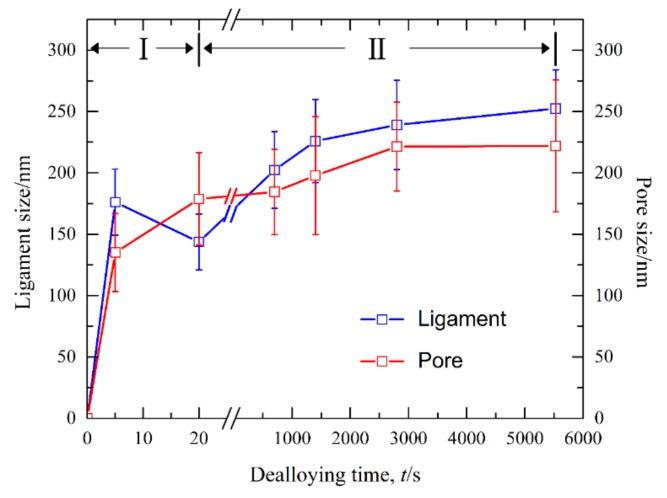
Mean sizes of the nanopores and ligaments after dealloying of Ag_40_Sn_60_ ribbons at −50 mV in 1.2 M HCl solution.

Figure 8 shows the current responses of Ag_40_Sn_60_ ribbons during potentiostatic dealloying at applied potential of 0, −50, and −100 mV in 1.2 M HCl solution. The curves were plotted on double logarithmic coordinates. The chronoamperometric curves show a typical dealloying process of dual-phase precursor alloys [27,37]. The current densities sharply fell from more than 400 mA cm^−2^ to about 5 mA cm^−2^ in the initial 20 s, which are consistent with the current densities at −0.1 V in Figure 4. It is considered that the selective dissolution of intermetallic phases in the subsequent periods went into a relatively steady stage. The higher the applied potential is, the higher the resulting current density in the steady stage is, indicating the higher the dissolution rates of Sn. In each case, the current densities quickly attenuated at the end of the response. It is noticed that the various trends of current response were consistent regardless of both the chemical compositions of the precursor alloys and applied potential, which suggest that an identical dealloying behavior for Ag–Sn alloys in this work is determined by the phase constitutions of ε-Ag_3_Sn and β-Sn phases in the matrix. It is worth noting that the transient curves of the dealloying currents for all Ag–Sn precursors are similar. On the basis of the transient characteristics of the dissolution currents of dual-phase Ag–Sn ribbons at different applied dealloying potential, the dealloying process is considered to divide into two stages: Stage I for the preferential dissolution of β-Sn phases at the beginning periods of about 20 s and Stage II for the continuous dissolution of ε-Ag_3_Sn phases to form the nanoporous silver. The potentiostatic dealloying of Ag_40_Sn_60_ ribbons at the potential of −50 mV was selected to investigate the dealloying behavior due to its suitable applied potential and dealloying duration. As shown in the insets of Figure 8, the Ag_40_Sn_60_ ribbon was bright silvery white and quickly became black after dealloying and gradually turned to silvery gray in Stage II. The micrograph of Ag_40_Sn_60_ ribbons before and after dealloying for different time is shown in Figure 9. Figure 9a–c shows the SEM images of Ag_40_Sn_60_ ribbon after potentiostatic dealloying at potential of −50 mV for 5 s. The top-view images in Figure 9a,b demonstrate that the nanoporous structure was partially formed on the surface of the ribbons instead of the whole surface. The cross-sectional image in Figure 9c further suggests bi-continuous nanoporous structure has not yet formed throughout the whole ribbons. After dealloying of 20 s, the uniform porous structure has formed in the surface. Meanwhile, the cross-sectional SEM images show that the porous structure has formed in the whole matrix, which is regarded to be resulted from the total dissolution of β-Sn phases in the matrix. Figure 10 shows the morphological evolution of nanoporous structures obtained by potentiostatic dealloying Ag_40_Sn_60_ ribbons at −50 mV for 700, 1400, and 2800 s in Stage II. The color change in the insets of Figure 8 also proved the change of the chemical composition of dealloyed ribbons. The EDX analysis has been performed on all as-dealloyed ribbons, and atomic ratio of Ag and Sn elements are summarized in Figure 11. The EDX analysis listed in Figure 11 determined the elemental composition of the ribbon after dealloying for 5 s were 63.37 at.% Ag and 36.63 at.% Sn, respectively. The XRD patterns of the series as-dealloyed ribbons are shown in Figure 12. The diffraction peaks corresponding to β-Sn phase in XRD pattern distinctly weakened after potentiostatic dealloying for 5 s. When the Ag_40_Sn_60_ ribbon was dealloyed for 20 s, as shown in Figure 9d–f, the uniform nanoporous structure was obtained across the whole section of the ribbons. The atomic percentage of Ag and Sn was confirmed to be close to 3:1 (specifically, 76.05 at.% for Ag atoms and 23.95 at.% for Sn atoms). Combined with the XRD pattern in Figure 12, it can be confirmed that the Ag–Sn ribbons were only composed of ε-Ag_3_Sn phases after dealloying of 20 s. The residual Sn in as-dealloyed ribbons decreased to 20.54 at.% after 700 s, 18.52 at.% after 1400 s and 7.39 at.% after 2800 s. As shown in Figure 12, with the dealloying process, the diffraction peaks corresponding to ε-Ag_3_Sn phase gradually weakened, while that corresponding to fcc Ag phase appeared and became gradually stronger. Additionally, the mean sizes of the ligaments and pores in as-dealloyed ribbons are summarized in Figure 13. The nanoporous structure obtained after dealloying for 5 s had a ligament size of 176 ± 27 nm and a pore size of 135 ± 32 nm, which is different from that obtained after dealloying for 20 s (ligament size was 144 ± 23 nm and pore size was 179 ± 38 nm). This change is due to the complete leaching out of the β-Sn phases. During the potentiostatic dealloying, the ligaments and pores increased in size to 202 ± 31 nm and 184 ± 35 nm after 700 s, 226 ± 34 nm and 198 ± 48 nm after 1400 s, 239 ± 36 nm and 221 ± 36 nm after 2800 s, and became as large as 252 ± 32 nm and 222 ± 54 nm. On the basis of above results, the dealloying of β-Sn solid solution and ε-Ag_3_Sn intermetallic are determined to be asynchronous in dual-phase Ag–Sn alloy. The sharp decrease in current responses mainly corresponds to the selective dissolution of the less noble β-Sn phases, and the steady stage is associated with the dealloying of the more noble ε-Ag_3_Sn phases.

## 4. Discussion

For the Ag–Sn alloy, the peak and plateau were observed on the potentiodynamic polarization curves in Figure 4. Similar polarization phenomenon has been reported in the previous researches [20,30]. Zhang [30] studied the polarization curves of Ag_20_Zn_80_ and Ag_30_Zn_70_ alloys in 5 wt.% HCl or 1 M NaCl solution and thought that two peaks and one plateau in curves may be correlated with the change of atomic ratios on alloy surface during potentiodynamic polarization process. Two plateaus and one peak were found in the polarization curve of amorphous Pd_42.5_Cu_30_Ni_7.5_P_20_ alloy in our former work, which were caused by the anodic dissolution or oxidation of Cu or Pd elements occurred at different potentials [20]. Obviously, the polarization behavior under anodic polarizations is closely related to the surface state of the electrodes. The polarization behavior of Ag–Sn alloys has been clarified by the phase evolution on alloy surface during potentiodynamic scanning process. When the potential was swept to −0.51 V, the β-Sn phase was forced to ionize as Sn^2+^ ions [38] and stripped of the outer surface into the electrolyte. The ε-Ag_3_Sn phases started to dissolve and transformed to fcc Ag phase as the potential was further swept to −0.46 V. It should be noted that the dissolution of β-Sn phase was dominant in the potential range of −0.46 V~−0.10 V due to low reaction rate of ε-Ag_3_Sn phases indicated by the polarization curves in Figure 4, although both phases dissolved. The decrease in current density from −0.24 V was attributed to the depletion of β-Sn phases on the outer surfaces of the precursor alloys based on the distribution profiles of β-Sn and ε-Ag_3_Sn phases in Figure 1 and Figure 2, the transient of the current densities during potentiostatic dealloying of Ag_40_Sn_60_ ribbon at applied potential of 0, −50, and −100 mV in 1.2 M HCl solution in Figure 8, the morphological change in Figure 9 and the change of phase distribution during dealloying from the XRD patterns in Figure 12. The occlusion by inert ε-Ag_3_Sn phases on surface prevented embedded β-Sn phases to dissolve. Additionally, the more inert Ag layer on ε-Ag_3_Sn surface prevented the dissolution of the latter. Because of the complete consumption of β-Sn phase on the outer surface, only ε-Ag_3_Sn phases were dissolved at lower rates, resulting in the current plateau at about 5~10 mA cm^−2^ in the potential range of −0.10 V~0.04 V corresponding to the anodic dissolution of ε-Ag_3_Sn phases. The increase in the current density at potential of higher than 0.04 V was caused by the anodic polarization of surface Ag-rich layer. The anodic dissolution of residual ε-Ag_3_Sn phase also took place in this potential region. The polarization behavior of Ag_20_Sn_80_ and Ag_30_Sn_70_ alloys can also be explained by the processes described above. Compared to the Ag_40_Sn_60_ alloy, the advantage of β-Sn phases in Ag_20_Sn_80_ and Ag_30_Sn_70_ alloys led to the higher peak current densities at about −0.24 V.

It is well established that the formation of nanoporous structure during dealloying involves the etching of the less noble metal by selective dissolution and the coarsening of the more noble metal by surface diffusion [9,11,18]. The results in Figure 5, Figure 6 and Figure 7 show that NPS can be obtained by potentiostatic dealloying of Ag–Sn alloys. According to the non-equilibrium solidification theory, primary ε-Ag_3_Sn phase first precipitated during rapid solidification of Ag_20_Sn_80_, Ag_30_Sn_70_, and Ag_40_Sn_60_ alloys, and then β-Sn/ε-Ag_3_Sn eutectic phase formed in the remaining liquids (Figure 1 and Figure 2). It should be noticed that the ε-Ag_3_Sn in the eutectic state is traced due to the very right eutectic point (96.2 at.% Sn) [33]. Therefore, the primary ε-Ag_3_Sn phase is considered to be surrounded by β-Sn phases. It has been confirmed that the dealloying of β-Sn and ε-Ag_3_Sn were asynchronous (Figure 9, Figure 10, Figure 11 and Figure 12). This asynchronous dealloying in dual-phase alloy is usually achieved with the greatest difference in electrochemical activities between coexisting phases [29,37]. As shown in Figure 3 and Figure 4, the electrochemical activity of β-Sn phases is much higher than that of ε-Ag_3_Sn. Thus, micro-coupling cells formed between β-Sn and ε-Ag_3_Sn phases in dual-phase precursor alloys in the free immersion conditions, in which β-Sn would act as an anode while ε-Ag_3_Sn would act as a cathode leading to a galvanic corrosion [10,29]. During the initial potentiostatic dealloying, β-Sn phases under the strong anodic polarized conditions were quickly excavated out of dual-phase Ag–Sn alloys due to its extremely high electrochemical activity. In Stage II, ε-Ag_3_Sn phases were conserved into as-dealloyed ribbons to form nanoporous Ag_3_Sn (NP-Ag_3_Sn) in the intermediate periods as shown in Figure 9d–f. The current densities at −50 mV in Table 1 and Figure 4 and dealloying currents in Figure 8 were very similar, which supports the division of the asynchronous dissolution of β-Sn phases in Stage I and ε-Ag_3_Sn phases in Stage II. The preferential dealloying phenomenon of the less noble phase in alloys were also found in dual-phase Mg_84.5_Cu_15.5_ precursors consisting of α-Mg and Mg_2_Cu phases and Al_85_Cu_15_ alloys with coexistence of both α-Al and Al_2_Cu phases in previous research [29]. With increasing dealloying time, ε-Ag_3_Sn phases in NP-Ag_3_Sn ribbons were further etched and gradually formed the NPS with uniform porous structure, as shown in Figure 10.

**Figure 14:**
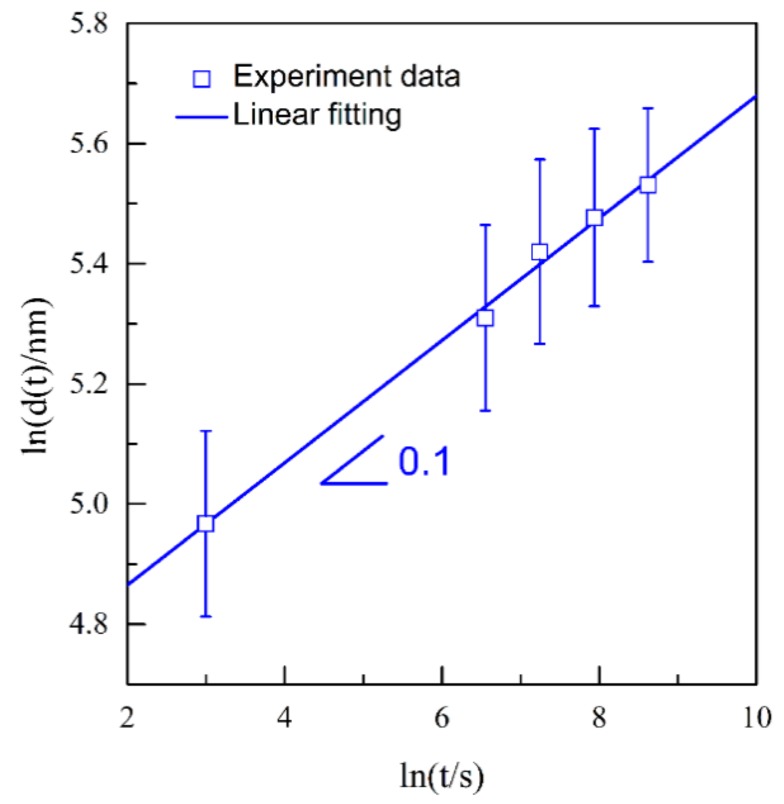
Double logarithmic plot of ln(*d*(*t*)) vs. ln*t* of Ag_40_Sn_60_ ribbons after dealloying at −50 mV in 1.2 M HCl solution.

The increase of nanostructures in size with time in Figure 13 shows a coarsening trend of nanoporous ligaments during the evolution from NP-Ag_3_Sn to NPS. In the dealloying of the Au–Ag system, Erlebacher [18] predicted that the characteristic length scales (*d*) of nanoporous gold (NPG) is the function of the surface diffusivity (*D_s_*) of Au atoms:

(1) $$d\propto {{(D}_{s}/V_{0})}^{\mu }$$

where *V*_0_ is the velocity of a flat alloy surface with no gold accumulated on it and *μ* is a constant. Qian and Chen [14] proposed the following relationship to predict the coarsening mechanism of nanoporous ligaments in dealloying:

(2) $${d(t)}^{n}{\;=\;\mathrm{KtD}}_{s}$$

where *D_s_* is the surface diffusivity of the nobler metal atoms, *K* is a constant, *t* is the dealloying time, *d*(*t*) is the ligament size at the dealloying time *t*, and *n* is the coarsening exponent. The coarsening exponent *n* can be directly measured by plotting ln[*d*(*t*)] vs. ln*t*, as shown in Figure 14. The result shows a dependency of ligament size on dealloying time as:

(3) $$d(t)\;\propto {\;t}^{0.1}$$

Thus, the *n* value for the coarsening of NPS is determined to be 0.1. The excellent linear relation between ln[*d*(*t*)] and ln*t* further verified the logarithmic coarsening mechanism of NPS. These facts indicate that the formation of the NPS is controlled by the surface diffusion of Ag adatoms. In the dealloying process of NP-Ag_3_Sn, the surface diffusion of Ag adatoms on ligaments are beneficial for the Sn atoms to be exposed to the electrolyte and then to be dissolved, leading to the coarsening of the existing ligaments. The coarsening exponent, 0.1, smaller than the theoretical value of 0.25, is considered to be due to the high electrochemical stability of ε-Ag_3_Sn phases as indicated in Figure 4. Based on the surface diffusion-controlled coarsening mechanism, the surface diffusivity (*D_s_*) of Ag adatoms along alloy/electrolyte interfaces can be estimated by the equation [11,14,30,34]:

(4) $$D_{s}\;=\;\frac{{d(t)}^{4}\mathrm{kT}}{{32\gamma t\alpha }^{4}}$$

where *k* is the Boltzmann constant (1.3806 × 10^−23^ J K^−1^), *T* is the absolute temperature (298 K), *γ* is the surface energy (1.302 J m^−2^) [39], and *α* is the lattice parameter of Ag (4.09 × 10^−10^ m). The calculated *D_s_* values with different alloy compositions and applied potentials are listed in Table 2. The present *D_s_* values of Ag adatoms are calculated to be 1.2 × 10^−12^ to 1.6 × 10^−10^ cm^2^ s^−1^, which are comparable the values (3.3 × 10^−12^ to 9.9 × 10^−11^ cm^2^ s^−1^) of Ag adatoms reported by Zhang [30] and are about 3 to 5 orders of magnitude greater than the value (2.0 × 10^−15^ cm^2^ s^−1^) of Au adatoms reported by Qian and Chen [14]. For the same alloys, the surface diffusivity of Ag adatoms increases with increasing applied potential from −100 mV to 0 mV. These results are in agreement with previous reports [30,34]. The influence of applied potential on the surface diffusivity of adatoms at the alloy/electrolyte interfaces has been explained as the consequence of an accumulation of charges on the electrode surface, which modifies the enthalpy of formation of surface moving entities and the formation of adsorbed bonds between neighboring sites due to electron transfer reactions [40,41]. Apart from the influence of applied potential, the adsorption of Cl^−^ ions can effectively accelerate the surface diffusion of the Ag adatoms during electrochemical dealloying in this work [28,31]. The high surface diffusivity of Ag adatoms eventually resulted in the formation of uniform NPS with unimodal porosity distributions during the electrochemical dealloying of NP-Ag_3_Sn ribbon. This is a great disparate with the NPG with bimodal pore distributions prepared by chemical dealloying Al–Au alloys, in which the excavation of the α-Al phases out of the alloy forms the large-sized pores and the dealloying of the Al_2_Au phase results in the small-sized pores on the walls of the large-sized ligaments [19]. If the dealloying parameters, such as dealloying potential, concentration of the dealloying solutions, and the introduction of the inhibitor agents for the surface diffusions are well adjusted, the bi-modal or ultrafine nanoporous silver can be achieved in future.

## 5. Conclusions

Nanoporous silver with a bi-continuous nanoporous structure were successfully fabricated through potentiostatic dealloying of dual-phase Ag*_x_*Sn_100−*x*_ (*x* = 20, 30, 40 at.%) alloys in 1.2 M HCl solution. Both open-circuit potentials and corrosion potentials of the dual-phase Ag–Sn alloys are mainly determined by the less noble β-Sn phase, rather than their chemical compositions. The open-circuit potential and corrosion potential of the β-Sn phase are lower than those of the ε-Ag_3_Sn phase by about 90 mV and 60 mV, respectively. The potentiodynamic polarization behavior of dual-phase Ag–Sn alloys are highly similar, independent of the chemical composition and volume fractions of two coexisting phases in the precursor alloys. The electrochemical dealloying of the β-Sn and ε-Ag_3_Sn phases is confirmed to be asynchronous. In the initial dealloying stages, the less noble β-Sn phases are preferentially etched due to high electrochemical activity and the galvanic corrosion. The subsequent dealloying of more noble ε-Ag_3_Sn phase is associated with the surface diffusion of Ag adatoms. Nanoporous ligaments become coarsened gradually with a time dependence of *d*(*t*) ∝ *t*^0.1^. The existing surface diffusivity of Ag adatoms are about 3 to 5 orders of magnitude greater than that of Au adatoms, which can be affected by the applied potential and halogen ions. This study not only develops a new alloy system for the preparation of NPS via potentiostatic dealloying, but also provides guidance for the design of dealloying process of similar alloy systems in the future.

## Figures and Tables

**Table 1:** Comparison of the open-circuit potentials (E_ocp_), corrosion potentials (E_corr_), and current densities (j_−50mV_) of Ag_20_Sn_80_, Ag_30_Sn_70_, and Ag_40_Sn_60_ alloys and Ag and Sn reference materials in 1.2 M HCl solution.

Alloys/Metals	Open-Circuit Potential (E_ocp_, mV)	Corrosion Potential (E_corr_, mV)	Current Density at −50 mV (j_−50mV_, mA cm^−2^)
Ag	−38	−111	0.0126
Ag_3_Sn	−407	−456	3.43
Ag_40_Sn_60_	−496	−513	7.34
Ag_30_Sn_70_	−498	−518	9.91
Ag_20_Sn_80_	−498	−515	5.00
Sn	−500	−513	397

**Table 2:** Summary of dealloying duration, ligament/pore sizes, and surface diffusivity of Ag adatoms during the potentiostatic dealloying of Ag–Sn alloys ribbons at the applied potential in 1.2 M HCl solution.

Alloy	Applied Potential, mV	Dealloying Duration, s	Ligament Size, nm	Pore Size, nm	Surface Diffusivity/*D_s_*, cm^2^ s^−1^
Ag_20_Sn_80_	−50	4673	182 ± 38	301 ± 49	8.3 × 10^−12^
Ag_30_Sn_70_	−50	4979	213 ± 33	249 ± 39	1.5 × 10^−11^
Ag_40_Sn_60_	0	1924	306 ± 50	295 ± 42	1.6 × 10^−10^
Ag_40_Sn_60_	−25	3611	278 ± 55	250 ± 34	5.8 × 10^−11^
Ag_40_Sn_60_	−50	5529	252 ± 32	222 ± 54	2.6 × 10^−11^
Ag_40_Sn_60_	−75	17,597	210 ± 32	190 ± 20	3.9 × 10^−12^
Ag_40_Sn_60_	−100	36745	190 ± 29	165 ± 39	1.2 × 10^−12^
